# A Feasibility Study of Personalized Prescription Schemes for Glioblastoma Patients Using a Proliferation and Invasion Glioma Model

**DOI:** 10.3390/cancers9050051

**Published:** 2017-05-13

**Authors:** Minsun Kim, Jakob Kotas, Jason Rockhill, Mark Phillips

**Affiliations:** 1 Department of Radiation Oncology, University of Washington, Seattle, WA 98195, USA; jkrock@uw.edu (J.R.); markp@uw.edu (M.P.); 2 Department of Mathematics, University of Portland, Portland, OR 97203, USA; jkotas@uw.edu

**Keywords:** radiotherapy treatment planning, glioblastoma, mathematical model

## Abstract

*Purpose:* This study investigates the feasibility of personalizing radiotherapy prescription schemes (treatment margins and fractional doses) for glioblastoma (GBM) patients and their potential benefits using a proliferation and invasion (PI) glioma model on phantoms. *Methods and Materials:* We propose a strategy to personalize radiotherapy prescription schemes by simulating the proliferation and invasion of the tumor in 2D according to the PI glioma model. We demonstrate the strategy and its potential benefits by presenting virtual cases, where the standard and personalized prescriptions were applied to the tumor. Standard prescription was assumed to deliver 46 Gy in 23 fractions to the initial, gross tumor volume (GTV_1_) plus a 2 cm margin and an additional 14 Gy in 7 fractions to the boost GTV_2_ plus a 2 cm margin. The virtual cases include the tumors with a moving velocity of 0.029 (slow-move), 0.079 (average-move), and 0.13 (fast-move) mm/day for the gross tumor volume (GTV) with a radius of 1 (small) and 2 (large) cm. For each tumor size and velocity, the margin around GTV_1_ and GTV_2_ was varied between 0–6 cm and 1–3 cm, respectively. Equivalent uniform dose (EUD) to normal brain was constrained to the EUD value obtained by using the standard prescription. Various linear dose policies, where the fractional dose is linearly decreasing, constant, or increasing, were investigated to estimate the temporal effect of the radiation dose on tumor cell-kills. The goal was to find the combination of margins for GTV_1_ and GTV_2_ and a linear dose policy, which minimize the tumor cell-surviving fraction (SF) under a normal tissue constraint. The efficacy of a personalized prescription was evaluated by tumor EUD and the estimated survival time. *Results:* The personalized prescription for the slow-move tumors was to use 3.0–3.5 cm margins for GTV_1_, and a 1.5 cm margin for GTV_2_. For the average- and fast-move tumors, it was optimal to use a 6.0 cm margin for GTV_1_ and then 1.5–3.0 cm margins for GTV_2_, suggesting a course of whole brain therapy followed by a boost to a smaller volume. It was more effective to deliver the boost sequentially using a linearly decreasing fractional dose for all tumors. Personalized prescriptions led to surviving fractions of 0.001–0.465% compared to the standard prescription, and increased the tumor EUDs by 25.3–49.3% and estimated survival times by 7.6–22.2 months. *Conclusions:* Personalizing treatment margins based on the measured proliferative capacity of GBM tumor cells can potentially lead to significant improvements in tumor cell kill and related clinical outcomes.

## 1. Introduction

Glioblastoma is highly heterogeneous, where different cells within the tumor respond to different types of therapy, leading to a treatment regimen involving multiple modalities [1]. Despite aggressive treatment with surgical resection and chemo-radiation, the prognosis remains poor with short median survival of less than 15 months with radiotherapy plus concomitant and adjuvant Temozolomide [2], and there has not been a dramatic improvement in survival over the last several decades [3].

Studies show that the extent of the surgical resection is an important prognostic factor in Glioblastoma Multiforme (GBM) [4]. However, most patients, including those who received the most radical surgical excisions, die of recurrent tumors [5]. Ogura et al. analyzed the locations of recurrent tumors for 21 patients [6]. Their study showed that initial recurrence occurred centrally at first (67% of the total patients) but distant recurrences were also observed in 19% of the patients. Cumulative recurrence patterns showed distant recurrences in the majority of the patients (88%). These clinical observations suggest that tumor cells spread throughout the brain. Swanson et al. published a mathematical model to describe the spatio-temporal dynamics of glioma cells using a reaction-diffusion equation [7]. Their model uses patient-specific, net rates of proliferation and invasion obtained from serial Magnetic Resonance Imaging (MRIs) as input parameters to describe how quickly the tumor grows and migrates in the spatiotemporal space. Therefore, their model predicts the tumor (both gross and microscopic disease) growth beyond what can be seen with current imaging technology. The prediction of tumor growth using their model also agrees with the clinical observation that glioma cells invade into normal brain. Therefore, the optimal extent of the treatment volume may affect clinical outcomes.

The standard treatment includes maximal safe resection followed by concurrent chemotherapy and radiotherapy to the resection cavity with a margin to eradicate the remaining tumor cells [8]. Radiation therapy of 60 Gy in 30 fractions is given either to one tumor volume or in two phases, where 46 Gy is given to a large volume and an additional 14 Gy to a reduced volume. The initial target volume consists of the gross tumor volume (GTV_1_), which is defined by either the T2 or Fluid-Attenuated Inversion Recovery (FLAIR) signal on the post-operative MRI scan, plus a margin. The boost target volume (GTV_2_) is based on the contrast-enhanced, T1 MRI scan, plus a margin [6]. A variable margin around GTV_1_ and GTV_2_ is currently used to define the clinical target volumes for each phase reflecting the difficulty in determining the exact extent of the tumor cells. Ghose et al. surveyed the variability in the practice patterns of Canadian radiation oncologists treating GBM [9]. They reported a significant variation in the margins used, ranging from 0.5 cm to 3.0 cm, with some of them using more than one standard margin. Multiple studies indicate that there is a lack of consensus in the most appropriate target volume to be treated [9,10,11].

The purpose of this study is to investigate the feasibility of personalizing prescription schemes using a tumor growth model, which includes patient-specific parameters, and to estimate their potential benefits on clinical outcome. Specifically, we assume that the spatiotemporal dynamics of the tumor follow the proliferation and invasion (PI) glioma model described in [12]. In this study, the prescription scheme is defined by the margins around GTV_1_ and GTV_2_, and the prescription dose for GTV_1_, while keeping the total dose for GTV_2_ to 60 Gy. We also investigated the temporal effect of the radiation on the tumor damage by linearly increasing or decreasing a fractional dose, and delivering a boost phase either concurrently or sequentially. The goal is to find the most effective treatment margins and temporal dose policy in killing tumor-cells without increasing the generalized equivalent uniform dose (gEUD) to normal brain compared to the current standard-of-care. The efficacy of the personalized prescription schemes was evaluated by comparing the cell-surviving fraction (SF), tumor EUD, and the estimated survival time with those of the standard-of-care. 

## 2. Methods and Materials

### 2.1. Prescription Geometry

**Figure 1:**
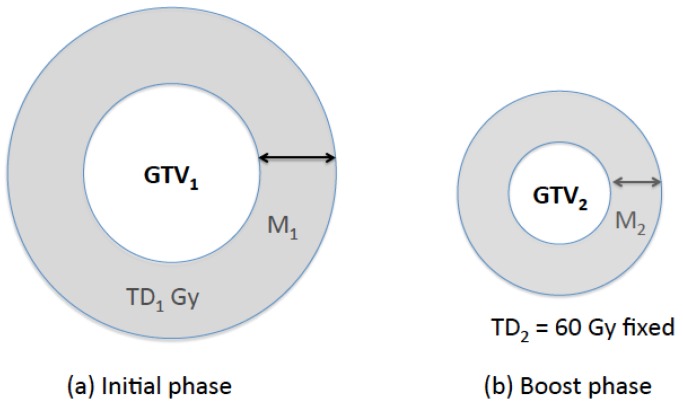
Prescription geometry. For the standard prescription, TD_1_ = 46 Gy, TD_2_ = 60 Gy, and M_1_ = M_2_ = 2 cm.

A standard prescription to treat GBM was assumed to deliver a total dose of 60 Gy (=TD_2_) in two phases, with 46 Gy (=TD_1_) being delivered in 23 fractions in the initial phase to GTV_1_ plus a 2cm margin followed by a boost of 14 Gy in 7 fractions to GTV_2_ plus a 2 cm margin (Figure 1) [11].

### 2.2. Tumor Growth Simulation Using a PI Glioma Model

We used the spatio-temporal model of the tumor proliferation and invasion under the effect of radiotherapy as presented in [12]. Let *D* be a diffusion coefficient, and *s* be the length of a radiotherapy session. Let *ρ* and *k* be the parameters related to the rate of cell proliferation and the carrying capacity of the tissue, respectively. Then the tumor dynamics under radiotherapy can be modeled as follows:

(1) $$\frac{\partial c}{\partial t}=\underset{\mathrm{Net}\;\mathrm{dispersion}}{\underset{︸}{\nabla \left(D\nabla c\right)}}+\underset{\mathrm{Net}\;\mathrm{proliferation}}{\underset{︸}{\rho c\left(1-c/k\right)}}-\underset{\begin{array}{c} \mathrm{Net}\;\mathrm{cell}-\mathrm{kills}\;\\ \mathrm{by}\;\mathrm{radiation} \end{array}}{\underset{︸}{Rc/s}}$$

where *c = c*(***x****, t*) is the glioma cell concentration and *R = R*(***x****, t*) is the cell loss due to radiotherapy at location ***x*** and time *t*. Equation (1) describes the rate of change in the glioma cell concentration at any given point in the brain in terms of the tumor dispersion velocity and proliferation rate, which vary among different patients and are measurable from two or more MRIs taken at different times. The linear quadratic (LQ) cell-survival model [13] gives the loss of glioma cells during radiotherapy as follows:

(2) $$R\left(x,t\right)=\;\{\begin{array}{c} 1-exp\left[-\alpha \left(d_{t}\left(x\right)+\frac{d_{t}\;{\left(x\right)}^{2}}{\alpha /\beta }\right)\right]:\;\mathrm{during}\;\mathrm{radiotherapy}\\ 0\;:\mathrm{between}\;\mathrm{fractions}, \end{array}$$

where *d_t_ (**x**)* is the dose administered at time *t* to location ***x***, and *α* and *β* are tissue specific radio-sensitivity parameters. We used the two-dimensional finite difference method to approximately solve for *c*(*x, y; t*) in Equation (1).

The tumor volume was simulated using Equation (1) with the following parameters as in [3]: *ρ* = 0.012/day, *α* = 0.035 Gy, *α/β* = 10 Gy, and *k* = 10^9^/cm^3^. Swanson et al. reported in [14] that the measured values of *D* from serial MRIs for 70 patients ranged from 0.24 to 35.92 mm^2^/day (mean 10.52, median 9.83 mm^2^/day) and used Fisher’s approximation to calculate an approximate radial velocity of expansion of the edge of a tumor (*v* = $\sqrt{2\;\rho D}$). The velocity (*v*) ranged between 0.0118 and 0.1438 mm/day. Based on this report, we used three values of *D* = 0.017, 0.13, and 0.34 mm/day to characterize slow-, average-, and fast-move tumors. 

Swanson et al. hypothesized that the circumference of the T2 MR hyper-intense area (GTV_1_) and T1-Gd MR hyper-intense area (GTV_2_) represent 16% and 80% of the maximum tumor cell concentration [15], respectively. We studied two different GTV_2_ sizes: GTV_1_ was obtained when the radius of GTV_2_ reached 1.0 and 2.0 cm from the initial condition of c_0_ (x_0_, y_0_; 0) = L^3^e^−100(x^2^_0_ + y^2^_0_)^, where L is the length of the computational domain [12]. The corresponding radius of GTV_1_ was recorded for each GTV_2_ (Table 1).

We simulated the tumor with and without modeling the resection cavity. The resection cavity was modeled by setting the number of tumor cells inside GTV_2_ to zero.

### 2.3. Prescription Scheme Variables

The goal is to find the treatment margins around GTV_1_ and GTV_2_ (i.e., M_1_ and M_2_) as well as TD_1_ delivered to GTV_1_ + M_1_, which leads to the minimum total number of tumor cells after 30 fractions. The margins M_1_ and M_2_ were chosen from the sets {0, 0.5, 1.0, ⋯, 6.0} and {1.0, 1.5, 2.0, 2.5, 3.0}, respectively. In our simulation, TD_2_ was fixed at 60 Gy. TD_1_ was constrained such that the generalized equivalent uniform dose (gEUD) of normal brain equals the gEUD resulting from the standard prescription. This means that the treatment with a larger margin is feasible only with a smaller dose to ensure that the toxicity of the non-standard prescription scheme on the normal brain does not exceed the toxicity from the standard prescription. Normal brain gEUD was calculated as follows [16]:

gEUD = (*f_1_*TD_1_^*a*^ + *f_2_*TD_2_^*a*^)^−*a*^, (3)

*f_1_ + f_2_* ≤ 1, TD_2_ = 60 Gy,

where *f_1_* and *f_2_* are the fractional areas irradiated to TD_1_ and TD_2_, respectively, and *a* is a tissue-specific sensitivity parameter. The sum of *f_1_* and *f_2_* would be less than 1 only if the irradiated area of the standard prescription is larger than the prescription scheme investigated, i.e., there is a fractional area (1–*f_1_ –f_2_*) with zero dose. TD_1_ is obtained by constraining gEUD of the normal brain to be the same as the standard prescription:

(*f_1_*^standard^ 46^*a*^ + *f_2_*^standard^ 6^0*a*^)^−*a*^ = (*f_1_^non-standard^*TD_1_^*a*^ + *f_2_^non-standard^* 60^*a*^)^−*a*^ (4)

**Figure 2:**
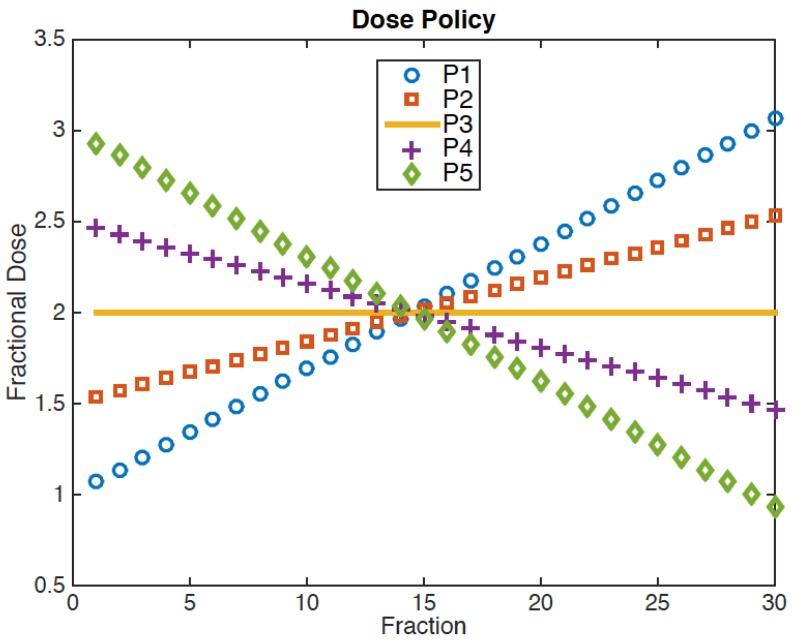
Non-stationary dose policies for the total dose of 60 Gy to GTV_2_+M_2_: P_1_/P_2_ and P_4_/P_5_ have a linearly increasing/decreasing fractional dose. P_3_ represents the constant dose per fraction as in the standard prescription.

Therefore, TD_1_ is a function of M_1_, M_2_, and the radii of GTV_1_ and GTV_2_. In this study, we used *a* = 5 for normal brain [15,17]. We also varied *a* between 3 and 7 to investigate the sensitivity of the optimal solutions on *a*. To examine the temporal effect of the radiation dose, five different, linear dose policies (P_1_–P_5_) have been implemented as shown in Figure 2. The sum of the fractional doses is fixed at 60 Gy. P_3_ represents the current, constant dose policy, in which the fractional dose is fixed at 2 Gy. Linearly increasing (P1/P2) or decreasing dose (P4/P5) policies were implemented with an initial dose of 1.0/1.5 Gy for P1/P2 and 2.5/3.0 Gy for P4/P5 (Figure 2). We ignored the fractionation effects on the normal brain because the linear dose policies investigated in this study result in a clinically insignificant difference in the biologically effective dose (BED) using *α*/*β* = 3 Gy, i.e., 100, 100.9, and 103.4 Gy for P3, P2/P4, and P1/P5, respectively. 

A boost phase was delivered (i) sequentially or (ii) concurrently. In the sequential boost, TD_1_ was delivered to GTV_1_ + M_1_ first with a fractional dose as shown in Figure 2, and then TD_2_−TD_1_ was additionally delivered to GTV_2_ + M_2_ only. Therefore, the number of fractions in the initial phase varied depending on TD_1_ and the dose policy used. In the concurrent boost, TD_1_ and TD_2_ were simultaneously delivered in 30 fractions to GTV_1_ + M_1_ and GTV_2_ + M_2_, respectively.

### 2.4. Evaluation Criteria 

The efficacy of each non-standard prescription scheme was evaluated by comparing the tumor cell-surviving fraction (SF) after 30 fractions relative to the SF that resulted from the standard prescription. In addition, the tumor EUD of the personalized prescription relative to the EUD from the standard prescription was calculated. The higher the EUD is, the higher the tumor cell-kill that is achieved. Let SF* and SF^0^ be the tumor cell-surviving fraction after the treatment course using the personalized prescription and the standard prescription, respectively. Similarly, let EUD* and EUD^0^ be the EUD from the personalized and the standard prescription, respectively. Then the surviving fraction can be written as:

$$SF^{*}=exp\left[-\alpha EUD^{*}-\beta {\left(EUD^{*}\right)}^{2}/N\right],\;SF^{0}=exp\left[-\alpha EUD^{0}-\beta {\left(EUD^{0}\right)}^{2}/N\right],$$

where *N* = 30 fractions. Therefore, EUD* as a function of EUD^0^ is given by:

(5) $$EUD^{*}=\left(\frac{N}{2\beta }\right)\left(-\alpha +\sqrt{\alpha^{2}+\frac{4\beta }{N}}\left(\alpha EUD^{0}+\frac{\beta {\left(EUD^{0}\right)}^{2}}{N}-ln\left(\frac{SF^{*}}{SF^{0}}\right)\right)\right).$$

EUD^0^ was calculated using Equation (3) with *a* = −10 for the tumor [17].

We also estimated the survival times for the standard and personalized prescriptions. Swanson et al. showed that their PI glioma model predicts the actual survival time by simulating the tumor growth until it reaches its fatal radius of 3 cm [18], which they used as an indicator of the interval to death. We let the tumor grow after the course of radiotherapy and measured the time required for GTV_2_ to reach a radius of 3 cm for the standard and personalized prescription schemes, respectively.

## 3. Results

Personalized prescription schemes were not affected by the following parameters: (i) modeling resection cavity in the tumor growth simulation (ii) radiobiological parameters (*α*/*β*) used in the LQ cell-survival model, and (iii) the EUD parameter, *a*. Therefore, all the results are presented in this section using *a* = 5 for the normal brain and *α/β* = 10 Gy for the tumor without the modeling resection cavity. In summary, Table 2 shows the personalized prescription scheme for each tumor size and velocity studied, which leads to the maximum cell-kills in the simulation. Table 3 shows the tumor EUD and the expected survival that results from using these personalized prescription schemes in Table 2. The percentage improvements are relative to the results from using the standard prescription.

### 3.1. Sequential and Concurrent Boost 

Tumor SF for the personalized prescription was normalized to that for the standard prescription using:

(6) $$\mathrm{Personalized}\;{\mathrm{SF}}^{*}\left(\%\right)=\frac{{\mathrm{SF}\;\mathrm{using}\;\mathrm{personalized}\;M}_{1},M_{2},\;{\mathrm{and}\;\mathrm{TD}}_{1}}{\mathrm{SF}\;\mathrm{using}\;\mathrm{the}\;\mathrm{standard}\;\mathrm{prescription}}\times 100.$$

Personalized SF (P-SF) using the sequential boost was 18.3–61.3% lower than the P-SF using the concurrent boost for all cases except for the fast-move, large tumor. In this case, the concurrent boost P-SF was 41.4% lower than the sequential boost P-SF. 

For the sequential boost, the personalized M_1_ ranged from 3.0–6.0 cm, and M_2_ was 1.5 cm for all tumors except for the fast-move, large tumor. The corresponding TD_1_ ranged between 20.7 and 45.6 Gy. A linearly decreasing dose policy P_5_, i.e., a larger fraction size upfront, led to the smallest SF for all tumor sizes and velocities. P-SF for all tumors studied was 0.001–0.465% of the SF that resulted from the standard prescription (Table 2).

### 3.2. Stationary Fractional Dose Policy

To evaluate if the constant fractional dose policy (P_3_) is clinically equivalent to the personalized dose policy, we compared SF using P_3_ to the standard prescription and to P_5_ (with M_1_, M_2_, and TD_1_ being fixed to their personalized values for both P_3_ and P_5_). For the different tumor characteristics (sizes and velocities), the SF using P_3_ relative to the SF using the standard prescription varied from 0.001–0.491% (sequential boost) and 0.002–0.330% (concurrent boost). Relative to P-SF, the stationary dose policy (P_3_) resulted in a higher SF: 105.7–218.8% (sequential) and 100.4–136.9% (concurrent).

### 3.3. Comparison of EUD and Estimated Survival Time

We computed EUD of the tumor using the personalized prescription (Table 2) relative to that with the standard prescription. (Note that EUD >100% indicates superiority of the personalized prescription; this is the opposite of the P-SF.) The EUD using the personalized prescription was 123.8–149.3% and 125.3–146.5% of the EUD from the standard prescription for the sequential and concurrent boost, respectively. The EUD using P_3_ and personalized M_1_, M_2_, and TD_1_ was 123.6–147.5% and 125.3–146.1% of the EUD resulting from the standard prescription for the sequential and concurrent boost, respectively. Therefore, the difference in the tumor EUD between P_5_ and P_3_ with a sequential boost is less than 3% for all tumors if M_1_, M_2_, and TD_1_ are fixed at their personalized values.

To calculate the effects of radiation therapy on the subsequent growth of the tumor, and hence the length of survival according to the model described in [7], the tumor cell distribution at the end of 30 fractions for each specific prescription was used as the starting point for calculating post-therapy tumor growth and spread. Then tumor growth was calculated according to Equation (1) with *R* = 0 (no radiation), and the time in months it took for the tumor to reach its fatal radius of 3.0 cm was determined. For the post-radiotherapy tumor growth, serial MRIs were not available to measure the patient-specific *ρ/D*. Therefore, we assumed that *ρ* was increased to 0.020/day to calibrate the survival time for the average-move tumors treated with the standard prescription to be approximately 19 months [19]. With this assumption, the optimal prescription led to a 7.6–22.2 months longer survival time than the standard prescription (Table 3).

## 4. Discussion 

In the past, many clinical trials have been conducted for the treatment of GBMs, but there has been little progress. The failures can occur at the edges of the radiation field and even in more distant locations in the brain. These patterns of failure have led to a current Phase II clinical trial to estimate the efficacy of low-dose whole brain irradiation (0.15 Gy per fraction to whole brain and 1.85 Gy per fraction to GTV for 30 fractions) to reduce the distant recurrence rate [20]. The growing understanding of tumor dynamics and proliferation, as exemplified by Swanson’s model [7], provides new impetus for exploring improved treatment strategies. 

Given the expense and time needed to carry out clinical trials, and given the good correspondence of the models with clinical data, we have an opportunity to provide some critical insight into some promising future directions. The purpose of our current study is to investigate the feasibility of personalizing the radiotherapy prescription scheme based on the individual tumor characteristics to increase the efficacy of tumor cell-kills using radiation. We utilized patient-specific, glioma dynamics developed and clinically validated by Swanson et al. [7]. Using their model, Rockne et al. studied the effect of the fractionation schedules, dose distribution, and radiation sensitivity parameters [12]. They concluded that hypofractionation is more effective than hyperfractionation.

Applying this model allows us to simulate the effects of tumor growth and spread of some prescription variables: treatment margins, fractional doses, and timing of boost irradiation. By applying dose limits on normal tissue and tumors that are currently accepted, we have limited our simulation to clinically feasible values. We limited the total prescription dose to 60 Gy since dose escalation beyond that point was not proven to be efficacious in improving clinical outcomes [21]. Our results indicate that individual tumor characteristics (velocity of growth, size) make a difference in the prescription scheme that leads to the minimum tumor SF (or equivalently maximum tumor EUD).

According to our study, the most significant factors in improving the treatment efficacy are the margins around GTV_1_ and GTV_2_. Although a linearly decreasing dose policy (P_5_), i.e., a larger fractional dose upfront, was found to be more effective than the constant fractional dose policy (P_3_), the difference from P_3_ was less than 3% in the tumor EUD. A sequential boost also leads to a lower SF than a concurrent boost for almost all tumor sizes and velocities; however, the EUD using the sequential boost was larger than the EUD using the concurrent boost by no more than 4%. For the average- and fast-move tumors, the personalized margin in the initial phase was 6.0 cm, which was the maximum margin used in this study. This suggests that whole brain radiotherapy in the initial phase to 20.7–38.3 Gy followed by the focal radiation to GTV_2_ plus 1.5 cm margin to 60 Gy (except for the fast-move, large tumors, where the optimal M_2_ is 3.0 cm) may be beneficial for patients with average- and fast-move tumors. On the other hand, it was found to be effective to use 3.0 and 3.5 cm margins in the initial phase to 43.7–45.6 Gy for slow-move, small, and large tumors, respectively, and then to use a 1.5 cm margin in the boost phase. Wernicke et al. investigated the effect of treatment margins for GBM and concluded that treating GBM with limited margins has been achieved without compromising overall survival or changing recurrence patterns [22]. As seen in Table 3, the expected survival varies significantly depending on the tumor size and velocity. Therefore, reporting the effect of treatment margins on survival without considering the individual tumor characteristics may lead to different conclusions. We also note that increasing treatment margins without modifying the prescription dose may increase normal tissue toxicity, which can also affect survival. 

The estimations of survival time (based on Swanson's empirical observation that the modeled tumor size correlates well with death [18]) indicate that personalizing the prescription scheme can potentially increase the survival time by up to 10, 16, and 22 months for slow-, average-, and fast-move tumors, respectively, even though we recognize that actual predictions of change in survival are difficult given the complex physiology involved. The largest difference in survival time between the personalized and standard prescription scheme was for the fast-move, small tumors. On the other hand, the improvement of using a personalized scheme was the least for the fast-move, large tumors.

## 5. Conclusions

This study proposes a method to personalize a prescription scheme tailored to the individual tumor characteristics, i.e., size and velocity as observed in serial MRIs. A different prescription scheme leads to the least SF (i.e., maximum EUD) for different tumor characteristics, which may explain the heterogeneous response to the same treatment among different patients. The results of this study show the potential benefit of using a PI glioma model to personalize prescription variables based on the individual tumor characteristics to improve clinical outcomes. Future work will include exploring various mathematical models and investigating the effect of the model on the personalized prescription schemes. For example, Eikenberry et al. used continuous diffusion-reaction equations to model the behavior of proliferation and migrating tumor cells, and their interactions with chemorepellents and the extracellular matrix, using stochastic transitions between migrating and proliferating glioma cells [23].

## Figures and Tables

**Table 1:** Tumor size and moving velocity used in this study.

Moving Velocity (mm/day)	GTV_1_ Radius (cm)	GTV_2_ Radius (cm)
0.029 (slow-move)	1.7	1.0 (small)
	2.7	2.0 (large)
0.079 (average-move)	2.7	1.0 (small)
	4.2	2.0 (large)
0.13 (fast-move)	2.7	1.0 (small)
	5.2	2.0 (large)

Gross tumor volume (GTV_1_) was simulated using the parameters of proliferation rate (ρ = 0.012 day^−1^) and carrying capacity (*k* = 10^9^ cm^−3^) for each size of GTV_2_.

**Table 2:** Personalized prescription schemes.

Velocity (mm/day)	GTV2 Radius (cm)	P-SF (%)	P-M_1_ (cm)	P-TD_1_ (Gy)	P-M_2_ (cm)	P-Boost Delivery	P-Dose Policy
0.029 (slow-move)	1.0	0.021	3.0	45.6	1.5	Sequential	P_5_
	2.0	0.048	3.5	43.7	1.5	Sequential	P_5_
0.079 (average-move)	1.0	0.001	6.0 (max)	37.0	1.5	Sequential	P_5_
	2.0	0.001	6.0 (max)	38.3	1.5	Sequential	P_5_
0.13 (fast-move)	1.0	0.001	6.0 (max)	37.0	1.5	Sequential	P_5_
	2.0	0.328	6.0 (max)	32.9	2.5	Concurrent	P_5_

Personalized (P-) treatment margins (M_1_ for GTV_1_ and M_2_ for GTV_2_), total dose in the initial phase (TD_1_), and dose policy are shown for each tumor studied. The tumor cell-surviving fraction (SF) using the personalized prescription is shown as a percentage of SF obtained from the standard prescription (M_1_ = M_2_ = 2 cm, TD_1_ = 46 Gy).

**Table 3:** Efficacy of personalized prescriptions.

Velocity (mm/day)	GTV2 Radius (cm)	P-Tumor EUD (%)	Std. Estimated Survival Time (Months)	P-Estimated Survival Time (Months)
0.029 (slow-move)	1.0	134.3	53.5	62.5
	2.0	130.4	47.4	57.3
0.079 (average-move)	1.0	149.3	20.6	36.9
	2.0	148.0	17.7	33.2
0.13 (fast-move)	1.0	147.9	19.6	41.8
	2.0	123.8	10.2	17.9

Personalized (P-) equivalent uniform dose (EUD) of the tumor relative to EUD using the standard prescription (=100%) and estimated survival time in months. Standard (Std.) survival time was estimated by calibrating a proliferation parameter to match with the published data [19].
